# Plasmacytoid myoepithelioma of minor salivary glands: report of case with emphasis in the immunohistochemical findings

**DOI:** 10.1186/1746-160X-7-24

**Published:** 2011-12-12

**Authors:** Esaú P Santos, Danielle RR Cavalcante, Allan UC Melo, José C Pereira, Margarete Z Gomes, Ricardo LC Albuquerque

**Affiliations:** 1 Department of Dentistry, School of Dentistry, University Tiradentes, Aracaju, SE, Brazil; 2 Department of Morphology, Faculty of Biology, University Tiradentes, Aracaju, SE, Brazil; 3 Department of Stomatology, School of Dentistry, University Tiradentes, Aracaju, SE, Brazil; 4 Department of Oral Surgery, School of Dentistry, University Tiradentes, Aracaju, SE, Brazil; 5 Department of Oral Pathology, School of Dentistry, University Tiradentes, Aracaju, SE, Brazil

## Abstract

Myoepithelioma is a rare benign tumor of the salivary glands and is usually seen in the parotid gland and the minor salivary glands. It was once considered to be a type of pleomorphic adenoma (PA), but myoepitheliomas are today believed to be relatively aggressive tumors. Myoepitheliomas are most common in young adults between the ages of 30 and 50 and there are very few cases reported in individuals less than 18 years of age. We report a case of myoepithelioma located in the hard palate in a 15-year-old Brazilian male. The tumor was composed of plasmacytoid myoepithelial cells. An analysis of the immunohistochemical profile of the tumor cells showed positivity for vimentin, S-100 protein, and glial fibrillary acidic protein (GFAP), but not for smooth muscle actin (α-SMA) and cytokeratin 14 (CK14). We report this case because of the rarity of this tumor, especially in adolescents. We also discuss the histological parameters of the differential diagnosis of this tumor as well as its immunohistochemical profile.

## Introduction

Myoepithelioma is believed to be a rare kind of salivary gland tumor. It was first described by Sheldon in 1943 and was then considered to be a variant of pleomorphic adenoma (PA) [1]. This tumor is usually located in the parotid gland and the minor salivary glands of the soft palate and represents less than 1% of all salivary gland tumors [2]. Several authors now consider this tumor as being a distinct pathological entity with a biological behavior different from that of mixed tumors, even though myoepithelioma was once considered to be a variant of PA with exclusively myoepithelial differentiation [3]. In fact, myoepitheliomas are believed to be more aggressive than PAs [4]. Based strictly on morphology, four distinct cellular components have been described: spindle, plasmacytoid (hyaline), epithelioid, and clear cells; a wide variety of combined or intermediate forms are also seen [3-5]. Nevertheless, it must be stressed that the myoepithelial nature has not been firmly established in most of these cell types, except for the spindle and some epithelioid cell types [6,7]. The stroma of these tumors is frequently composed of fibro-hyalinized or myxoid connective tissue, similar to that seen in some PAs; however, in contrast to the latter, myoepitheliomas do not present chondroid or osteoid formation. Besides, ductal/luminal differentiation is not normally expected in myoepithelioma and, when present, it constitutes less than 5% of the tumor parenchyma; this is quite useful for distinguishing this lesion from a mixed tumor [7].

The majority of cases of myoepithelioma present as painless, slowly growing, firm masses, usually of small size. The biggest series published to date included 23 cases of myoepithelioma, with none affecting patients less than 18 years of age [8].

We present a case of plasmacytoid myoepithelioma (PM) in the hard palate of a 15-year-old adolescent. The histological parameters of the differential diagnosis, cellular phenotype differentiation pattern, and terminology are also discussed.

## Case report

**Figure 1:**
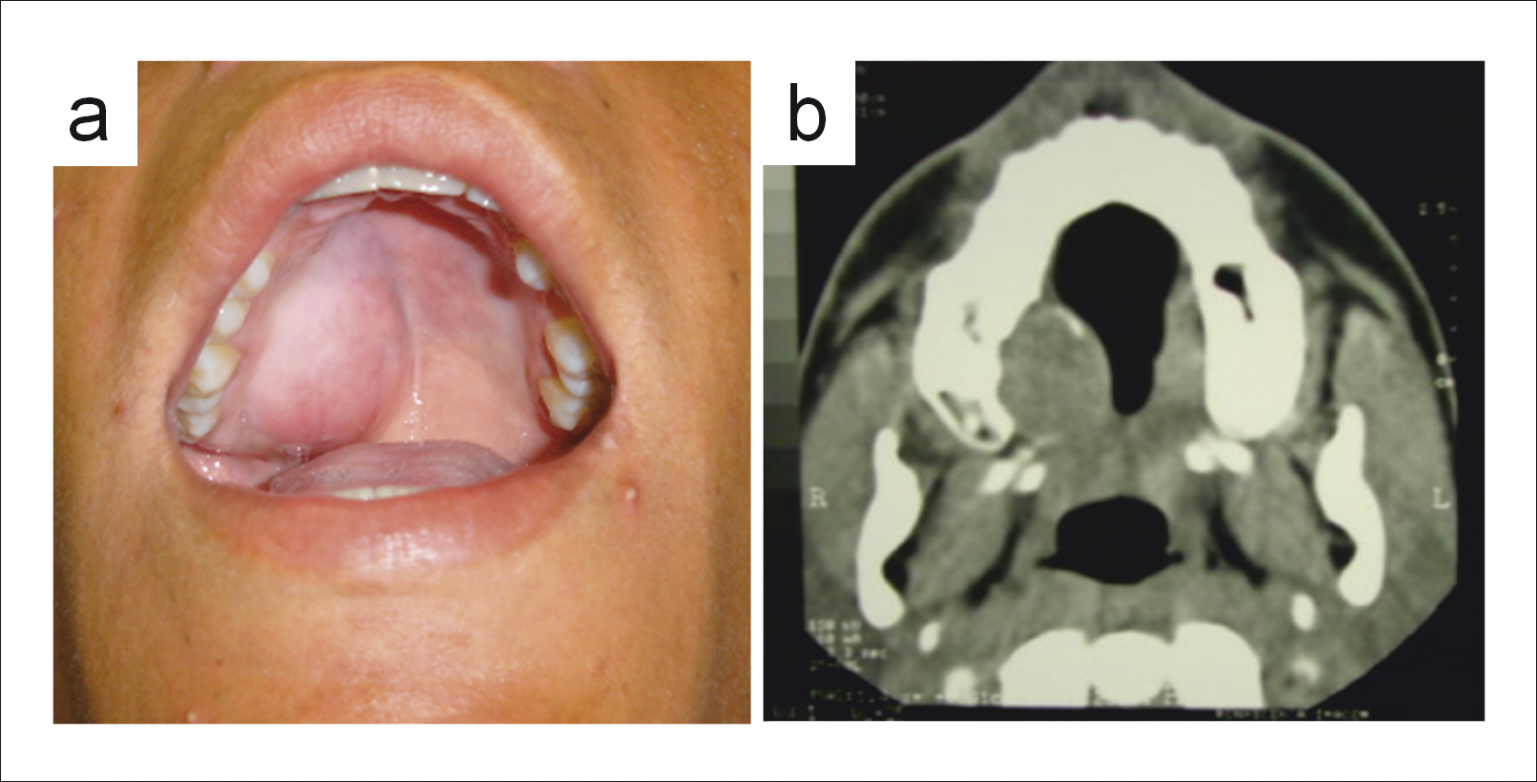
**Clinical and imaging features**. (a) Intraoral swelling in the right side of the hard palate. (b) Computed tomography showing large hypodense tumor provoking slight erosion of maxillary cortical bone.

A 15-year-old male who complained of a swelling inside his mouth was referred to the Oral Diagnosis Service of the School of Dentistry of the University Tiradentes (Aracaju/SE, Brazil) in March of 2005. Intraoral examination revealed an asymptomatic swelling on the right side of the hard palate. It was approximately 3.5 cm in size and according to the patient had been present for the past 1 year. The overlying mucosa was intact and normal in color and appearance. The teeth involved were all vital and no phlogistic signs were observed (Figure 1a). His past medical history and the family history were analyzed in detail but were noncontributory. Computed tomography of the lesion was done and showed a large hypodense tumoral image in the right side of the palate, where it had provoked a slight erosion of the maxillary cortical bone (Figure 1b).

**Figure 2:**
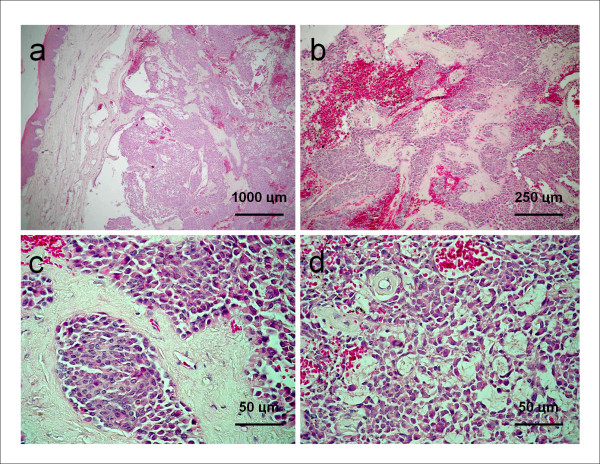
**Histopathology findings**. Histological sections stained in HE. (a) Well-circumscribed proliferation of sheets, islands, and strings of myoepithelial cells (40×); (b) Strong hyalinization of the connective tissue and foci of hemorrhage seen amidst the tumoral cells (Hematoxyin/Eosin, 40×) (c) Detail of the round-shaped myoepithelial cells showing eccentric nucleus (100×); (d) Tumoral plasmacytoid cells arranged in a pseudo-cribriform pattern within myxomatous background (100×).

**Figure 3:**
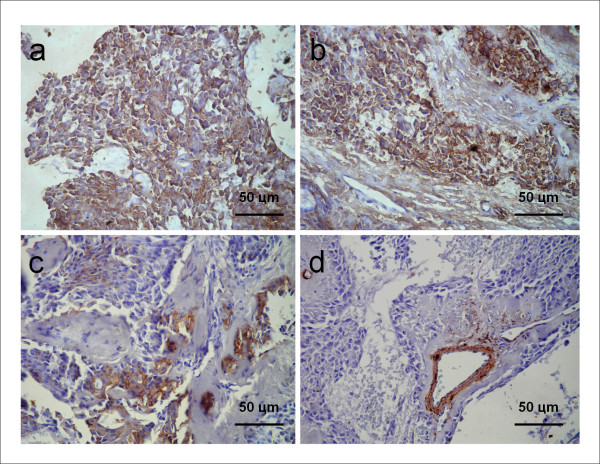
**Immunohistochemical findings**. Immunohistochemical study of tumor plasmacytoid cells revealed (a) strong positivity for S-100 protein, (b) moderate immunoreactivity for vimentin, and (c) focal immunoreactivity for GFAP, (d) Tumor cells failed in immunostaining for α-SMA, although the muscular walls of the blood vessels (positive control) were positive (Streptavidin-Biotin Complex, 100×).

Incisional biopsy was performed and the surgical specimen was sent for histopathological analysis. Histological sections of the specimen revealed a salivary gland neoplasm whose parenchyma consisted of plasmacytoid cells with eccentric round nuclei and eosinophilic (hyaline) cytoplasm, predominantly arranged in islands and sheets of tumoral cells. Less commonly, the tumoral cells were organized in anastomosing strings mimicking a pseudo-cribriform arrangement. Foci of hemorrhage and hemosiderin pigmentation were also found. The stroma showed strong hyalinization of the connective tissue with focal areas of myxoid changes (Figure 2). Immunohistochemically, the cytoplasm of the plasmacytoid cells was positive for S-100 protein and vimentin (Figures 3a and 3b) and negative for smooth muscle actin (α-SMA) (Figure 3d) and cytokeratin 14 (CK14). Focal reactivity for glial fibrillary acidic protein (GFAP) was observed in some areas (Figure 3c). The overall picture suggested the diagnosis of PM. The definitive treatment in this situation was surgical excision, extending down to the periosteum and including the overlying mucosa. The patient continues to be under rigorous follow-up and no recurrence has been detected so far.

## Discussion

Myoepitheliomas are benign neoplasms of salivary glands derived from myoepithelial cells. These tumors can occur at any age but are most common in young adults between the ages of 30 and 50, with the average of age in 36.3 years [3]. Most myoepitheliomas affect the parotid gland and minor salivary glands of the palate [2,7]. At date, and in our knowledge, it has been reported seven cases of plasmacytoid myoepithelioma from palate in children or adolescents [9-14], including the present one (table 1), attesting the rarity of this tumor in young patients.

**Table 1:** Demographic data of PM of palate occurring in younger reported in literature (Including present case).

Authors	Age	Gender
Kahn & Schoub (1973)	17	Female
Nesland et al (1981)	18	Female
Lins & Gnepp (1986)	8	Female
Arkuszewski P et al (2005)	12	Male
Nwoku et al (2005)	11	Male
Perez et al (2007)	13	Male
Santos et al (2011)	15	Male

In the current case, the tumor presented as a well-defined homogeneous enhancement with smooth contour. This CT pattern has been previously reported in benign myoepitheliomas of the palate [15]. However, slight erosion of the maxillary bone was observed in this case. Although the bone involvement might be interpreted as imaginologic signs of malignancy [16], erosion of the palatal cortical bone has also been seen in other benign salivary gland tumors of the palate, such as pleomorphic adenomas [17].

Although the architectural variations of myoepitheliomas are well defined, it must be emphasized that they can at times be difficult to differentiate from other tumors, particularly PAs. It has been suggested that these lesions are two different forms of the same entity [7]. However, other authors have stressed that myoepitheliomas are tumors exclusively composed of myoepithelial cells, with an absent or inconspicuous ductal component, and must be definitely differentiated from mixed tumors as they may present a more aggressive behavior [18]. In the present case, the neoplastic cells were all round-shaped with eccentric nuclei and eosinophilic hyalinized cytoplasm and thus resembled plasma cells. No ductal/luminal cellular differentiation was seen in either the incisional or excisional surgical specimens. These findings are in agreement with the reports in literature and are absolutely consistent with the diagnosis of PM [2,14,19,20]. In addition, despite the fact that this tumor showed intense hyalinization of the connective tissue as well as foci of myxoid changes, no evidence of chondroid or osteoid tissue was found. Similar findings were described by other authors [21], who emphasize that this sort of stromal differentiation is to be expected in PAs but not in myoepitheliomas. The pseudo-cribriform pattern seen in some focal areas of the tumor could perhaps lead to a misdiagnosis of adenoid cystic carcinoma. However, in contrast to myoepitheliomas, these last malignant tumors are clearly infiltrative and, characteristically, show basal membrane-like globules surrounded by rather bland myoepithelial cells with hyperchromatic nuclei, arranged in tubular, cribriform, and solid patterns [3].

It is also important to separate benign from malignant variants of myoepitheliomas. Malignant tumors are differentiated from their benign counterparts by their characteristic multi-lobulated architecture, presence of infiltrating growth, necrotic areas, cellular polymorphism, and mitotic figures [19]. Since none of these histological features were observed in this case, in addition to the lack of cell atypia, it was considered as a typical benign neoplasm. It has also been suggested that assessment of cell proliferative activity may be helpful in the differential diagnosis between benign and malignant myoepitheliomas. In this vein, a Ki-67 labelling index of more than 10% in myoepitheliomas is highly suggestive of malignant biological behavior [8].

The immunohistochemical study of the current case demonstrated positivity for S-100 protein and vimentin but not for α-SMA and CK14. Focal positivity was also seen for GFAP. Normal myoepithelial cells show myogenic differentiation, which is revealed by the presence of actin filaments as well as filaments of cytokeratin. However, tumoral myoepithelial cells rarely show the same cytoskeleton as normal cells, although some traces of the normal components of the cytoskeleton may be retained. Therefore, it is suggested that tumor myoepithelial cells might exhibit different stages of differentiation [21,22].

CK14 is responsible for the anchorage of myoepithelial cells to the basement membrane and is considered a useful marker of normal myoepithelial cells; it is usually unexpressed in tumor cells, unless those cells present terminal differentiation [23]. Once the myoepithelial cells in myoepitheliomas are no longer confined to the basement membrane -- which is fragmented in these tumors -- lack of CK14 expression is supposed to be expected [19]. In the current case, these findings were confirmed, as the tumor cells showed no reactivity for this particular cytokeratin.

The negativity for myogenic markers is expected in the plasmacytoid variant, a quite rare and controversial subtype of myoepithelioma that frequently lacks myogenic differentiation, even though it is positive for pan-cytokeratin [24,25]. It has been demonstrated that cultured cell lines derived from PMs express α-SMA, but this is not so for the tumoral cells of paraffin-embedded tissue. These findings suggest that plasmacytoid cells show full myoepithelial differentiation *in vitro*. Thus, they should be considered myoepithelial-like cells, and the lack of myogenic differentiation *in vivo *could be due to an inhibitory process mediated by the extracellular matrix [24]. Supporting this theory, the neoplastic cells were negative for α-SMA In the present case. On the other hand, immunopositivity for myogenic markers in PM has been demonstrated by Scarpellini *et al*., [25] suggesting that these plasmacytoid cells might exhibit distinct myoepithelial phenotypes in different tumors.

Immunoreactivity for S-100 protein is currently considered an important characteristic of this morphologic variant of myoepithelioma [3]. Similar to the findings in the present case, many studies have reported strong S-100 positivity in this kind of salivary gland tumor [3,8,14,20]. On the other hand, some authors have asserted that these plasmacytoid cells are also positive for GFAP [19], which was confirmed in the current case. Moreover, vimentin was also expressed by tumoral plasmacytoid cells in this case. Although this antigen is frequently detected in mesenchymal cells, in this case, it was extensively expressed in the neoplastic myoepithelial cells [19,26,27]. It is suggested that the immunohistochemical expression of vimentin may indicate that myoepithelial cells in tumors such as myoepitheliomas do not reach complete differentiation [24].

Despite the fact that some studies have pointed to a myoepithelial nature for plasmacytoid cells, studies have provided some evidence that plasmacytoid cells might present a luminal phenotype in PAs, as long as they failed in expressing myogenic markers, such as α-SMA, but widely expressed CKs 18 and 19 [6]. This particular profile is expressed by the luminal cells and some of the basal cells of normal salivary glands [24]. Nevertheless, further studies are necessary to find out whether plasmacytoid cells in myoepithelioma are true myoepithelial cells or not.

As performed in the current case, surgery with a margin of normal uninvolved tissue being included within the surgical excision is the first choice of treatment for benign myoepitheliomas, and the recurrence rates are similar to those of the pleomorphic adenomas [15]. The prognosis for benign myoepitheliomas is quite favorable, but patients should undergo regular follow-up examinations to rule out local recurrence [18].

## Consent

Written consent for publication was obtained from the patient's parent.

## Competing interests

The authors declare that they have no competing interests.

## Authors' contributions

SEP and AJRLC drafted the manuscript. AJRLC, GMZ, SEP and CDRR carried out the histological analysis, wrote the histological part of the paper and contributed to the writing of the final version. PJC, MAUC and SEP analysed the patient's history, reviewed the patient data and surgically removed the tumors. Each author reviewed the paper for content and contributed to the writing of the manuscript. All authors approved the final report.
